# Islands Containing Slowly Hydrolyzable GTP Analogs Promote Microtubule Rescues

**DOI:** 10.1371/journal.pone.0030103

**Published:** 2012-01-17

**Authors:** Carolina Tropini, Elizabeth A. Roth, Marija Zanic, Melissa K. Gardner, Jonathon Howard

**Affiliations:** 1 Marine Biological Laboratory Physiology Course, Woods Hole, Massachusetts, United States of America; 2 Max Planck Institute of Molecular Cell Biology and Genetics, Dresden, Germany; 3 Department of Genetics, Cell Biology, and Development, University of Minnesota, Minneapolis, Minnesota, United States of America; 4 Biophysics Program, Structural Biology Department, Stanford University, Stanford, California, United States of America; 5 Molecular Biology Interdepartmental Ph.D. Program, University of California, Los Angeles, California, United States of America; Virginia Tech, United States of America

## Abstract

Microtubules are dynamic polymers of GTP- and GDP-tubulin that undergo stochastic transitions between growing and shrinking phases. Rescues, the conversion from shrinking to growing, have recently been proposed to be to the result of regrowth at GTP-tubulin islands within the lattice of growing microtubules. By introducing mixed GTP/GDP/GMPCPP (GXP) regions within the lattice of dynamic microtubules, we reconstituted GXP islands *in vitro* (GMPCPP is the slowly hydrolyzable GTP analog guanosine-5′-[(α,β)-methyleno]triphosphate). We found that such islands could reproducibly induce rescues and that the probability of rescue correlated with both the size of the island and the percentage of GMPCPP-tubulin within the island. The islands slowed the depolymerization rate of shortening microtubules and promoted regrowth more readily than GMPCPP seeds. Together, these findings provide new mechanistic insights supporting the possibility that rescues could be triggered by enriched GTP-tubulin regions and present a new tool for studying such rescue events *in vitro*.

## Introduction

Microtubules are self-assembling, dynamic polymers that have essential functions in eukaryotic cell structure maintenance, cell division, intracellular transport and other processes [1]. Microtubules are composed of tubulin dimers that polymerize head-to-tail to form a protofilament; thirteen of these protofilaments associate laterally to form the cylindrical wall of the microtubule [1]. Tubulin dimers add on to a growing microtubule in the GTP-bound form and subsequently undergo hydrolysis to form GDP-tubulin [1], [2]. GDP-tubulin is more unstable than GTP-tubulin and more likely to depolymerize [1]–[3]. The growing end of a microtubule is therefore thought to maintain a GTP-tubulin cap that stabilizes it against depolymerization [2], [4]. Loss of the GTP-tubulin cap leads to a rapid shrinking phase; the conversion from growth to shrinkage is referred to as a catastrophe [1]–[4].

Islands of non-hydrolyzed GTP-tubulin left behind in the microtubule lattice during microtubule growth have recently been proposed to trigger the conversion of a shortening microtubule to a growing one [4], [5]. According to this proposal, such conversions—termed rescue—occur when the depolymerizing microtubule end reaches the island [4], [5]. Evidence for this mechanism originates from post-rescue observations of fixed microtubules using an antibody raised against GTPγS- bound tubulin: locations of rescue sites co-localize with microtubule areas recognized by the antibody [4]. The GTP islands have been suggested to act as depolymerization “speed bumps” or “stop signs,” slowing down or stopping depolymerization altogether and allowing the microtubule to begin regrowth [4], [5]. Recent results also suggest that GTP regions are dynamic structures that allow for rapid tubulin subunit on-off kinetics [6], supporting the general notion that GTP-rich regions could facilitate microtubule regrowth.

## Results and Discussion

**Figure 1:**
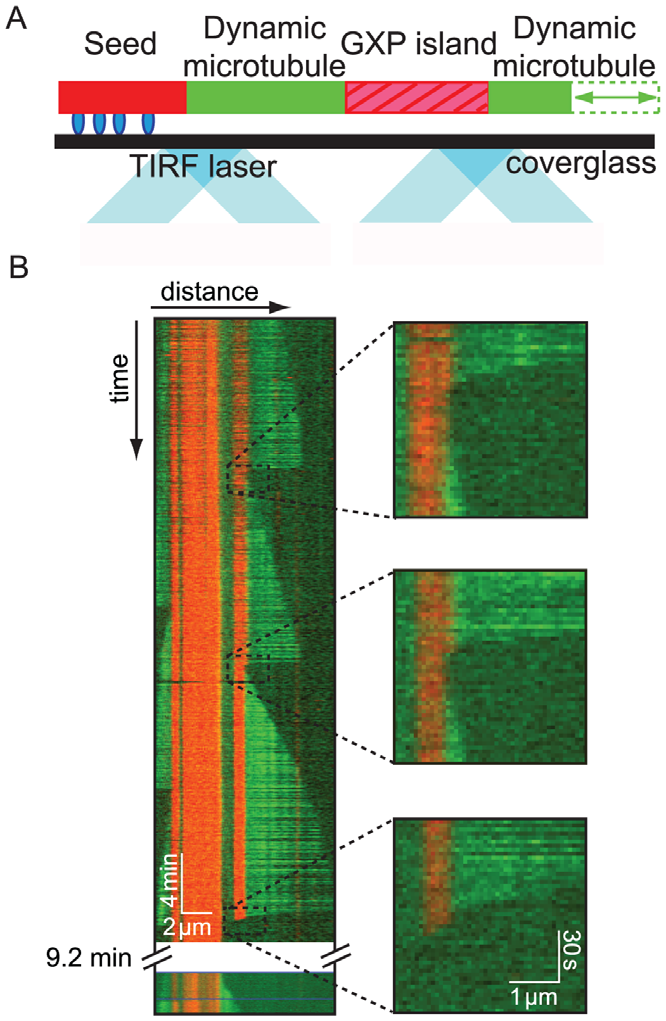
GXP (GTP/GDP/GMPCPP) islands promote microtubule rescue. (A) Schematic of the total internal reflection fluorescence (TIRF) microscopy setup for incorporating rhodamine-labeled GXP islands into alexa-488-labeled microtubules. (B) Kymograph of microtubule growth and depolymerization taken from time-lapse microscopy (Movie S1). Three types of rescue events were observed at GXP islands: (top right) rescue at the end of the GXP island, in which islands exhibited no detectable depolymerization; (middle right) rescue in the middle of the GXP island, in which islands partially depolymerized prior to re-growth; (bottom right) catastrophe through the island, in which the GXP island completely depolymerized.

To test the hypothesis that GTP islands rescue microtubule growth, we used the slowly hydrolyzable GTP analog GMPCPP (guanosine-5′-[(α,β)-methyleno]triphosphate) [7] to mimic the non-hydrolyzed GTP leftover in islands within the lattices of dynamic microtubules (Fig. 1A). The ends of microtubules assembled with GTP can be transiently stabilized against dilution-induced disassembly by capping the ends with GMPCPP-tubulin subunits [8]–[10]. Instead of capping the microtubule end in our experiments, we used GMPCPP-tubulin to introduce “GXP islands” at specific locations within the microtubule lattice. These GXP islands are defined as lattice regions containing a mixture of GTP-, GDP-, and GMPCPP-tubulin subunits and were identified by a different fluorescent tubulin label.

### GXP Islands Promote Rescue

To create GXP islands, we first introduced alexa-488-labeled GTP-tubulin into a flow channel in which rhodamine-labeled GMPCPP microtubule seeds had been bound to the cover-slip. Under these conditions, green GTP-tubulin microtubule extensions grew readily from the red seeds. After ∼1.5 minutes of growth time, a mixture of red rhodamine-labeled GMPCPP- and GTP-tubulin was introduced into the flow chamber to allow red GXP islands to grow from the green GTP-tubulin microtubule ends (Fig. 1A). After a brief reaction time of ∼1 minute, the red tubulin was flushed from the flow chamber, and the green GTP-tubulin was reintroduced (see Methods for further details). A low concentration of the microtubule plus-end binding protein EB1 was also added to increase the frequency of catastrophe events during each experiment [11]. Because EB1 does not bind the ends of depolymerizing microtubules and is expected to bind only weakly to the GMPCPP containing islands under our assay conditions [12], we do not expect the EB1 to affect the interaction of depolymerizing microtubules ends with the GXP islands.

Green GDP-tubulin microtubule extensions grew until a catastrophe event occurred, leading the microtubules to depolymerize into the red GXP islands (Fig. 1B and Movie S1). Three different outcomes were observed when depolymerizing green extensions reached the red GXP islands (Fig. 1B). In 58% of the cases (52 events), the microtubules rescued without any detectable depolymerization of the GXP island; a new green GDP-tubulin extension subsequently grew from the intact GXP island (Fig. 1B, top right). In 26% of the cases (23 events), microtubule depolymerization did not immediately cease at the end of the GXP island. Instead, the microtubule depolymerized partially into the GXP island before rescuing and starting to grow again (Fig. 1B, middle right). Finally, in 17% of the cases (15 events), no rescue event occurred, and the microtubule depolymerized through the entire GXP island (Fig. 1B, bottom right). No rescue in the GDP-tubulin extension region was observed.

These results show that model GXP islands that promote rescue, can be reconstituted *in vitro*. This supports the hypothesis that microtubule rescues are triggered by regions of enriched GTP-tubulin in the lattice. Importantly, because GTP binds tubulin with higher affinity than GMPCPP [7], [10], [13], the actual concentration of GMPCPP-tubulin in the GXP islands is predicted to be 42%, with the remaining 58% being GDP-tubulin (based on 74% GMPCPP and 26% GTP introduced into the flow channel and the radionucleotide incorporation assay from [10]; see Information S1 for more details). Assuming that GMPCPP-tubulin is randomly distributed throughout islands, we expect that a depolymerizing microtubule extension will encounter an average of 5.5 GMPCPP-tubulin-capped protofilaments within a 13-protofilament GXP island (we are neglecting the fact that GMPCPP-tubulin microtubules contain mainly 14 protofilaments [14], and that therefore the average number of protofilaments is likely to be between 13 and 14; this will make little difference to our argument). A high variability is expected: assuming random incorporation of tubulin subunits, the SD = √5.5, and as many as 10 GMPCPP-tubulin-capped protofilaments are expected to be encountered during depolymerization of an island of length 320 nm (assuming a normal distribution, 2.5% of the 13-protofilament caps will have 5.5+1.96√5.5 GMPCPP subunits). In our experiments, most, but not all, shrinking microtubules rescued without any detectable depolymerization of the island. This observed variability suggests that a threshold of capped protofilaments, perhaps as many as 10, is required for a rescue event to occur. Microtubules may depolymerize until an adequate number of GMPCPP-tubulin-capped protofilaments are encountered to stop shortening long enough for microtubule growth to resume.

### Rescue Events Correlate with the Size and Composition of the Islands

**Figure 2:**
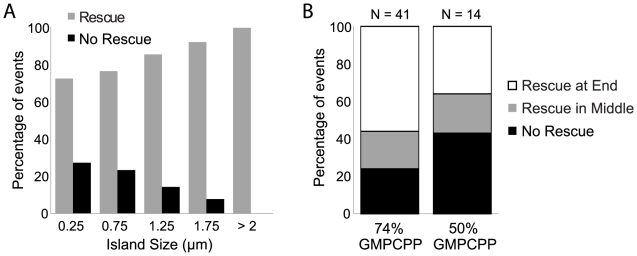
Island size and composition affect rescue efficiency. (A) The probability of rescue at either the middle or end of an island increased with the size of the island (see Table S1 for details). (B) Islands with a higher GMPCPP content (74% GMPCPP, island length ≤1 µm; 24% no rescue, 20% rescue in the middle of the island, 56% rescue at the end of the island; *N* = 41) had a higher probability of rescue than islands with a lower GMPCPP content (50% GMPCPP, island length ≤1 µm; 43% no rescue, 21% rescue in the middle of the island, 36% rescue at the end of the island; *N* = 14). See Table S1 for islands of all sizes.

If rescue events require some threshold number of GMPCPP-tubulin subunits to be encountered within a GXP island, rescue efficiency should be influenced by changes in the size or nucleotide composition of the island. We observed that the probability of rescue increased with the size of the island (Fig. 2A). The number of cases in which islands completely depolymerized (black histogram in Fig. 2A) decreased steadily from 27% for small <0.5 µm islands to 0% for >2 µm islands (*N* = 90 total events, up to 40 minutes of observation). Therefore, increased island size resulted in higher rescue probability, consistent with the hypothesis that GXP islands may depolymerize until an enriched GMPCPP-tubulin region is exposed; a longer island has increased probability of having several neighboring capped protofilaments and thus has an increased probability of rescue.

To test whether the nucleotide composition of the island would also affect the probability of rescue, we introduced 50% GMPCPP-tubulin (rather than 74%) into the flow chamber during the island polymerization step. In this case, it is predicted that 21% of the island lattice contains GMPCPP-tubulin subunits, while the remaining 79% of the island subunits can hydrolyze GTP to GDP (see above). Indeed, the frequency of rescue events inside similarly sized islands (less than 1 µm in length) was reduced for these 50% GMPCPP islands compared to previous experiments using 74% GMPCPP (Fig. 2B). Complete shortening through 50% GMPCPP islands was also more likely to occur than rescue within the island (Fig. 2B). These experiments may therefore span a critical threshold for rescue efficiency that could correspond to the minimum number of GMPCPP-tubulin-capped protofilaments that are required to efficiently promote rescue. Further experiments will be required to fully characterize the upper and lower bounds of GMPCPP concentrations required for rescue.

### GXP Islands Show Distinct Shortening and Regrowth Dynamics

**Figure 3:**
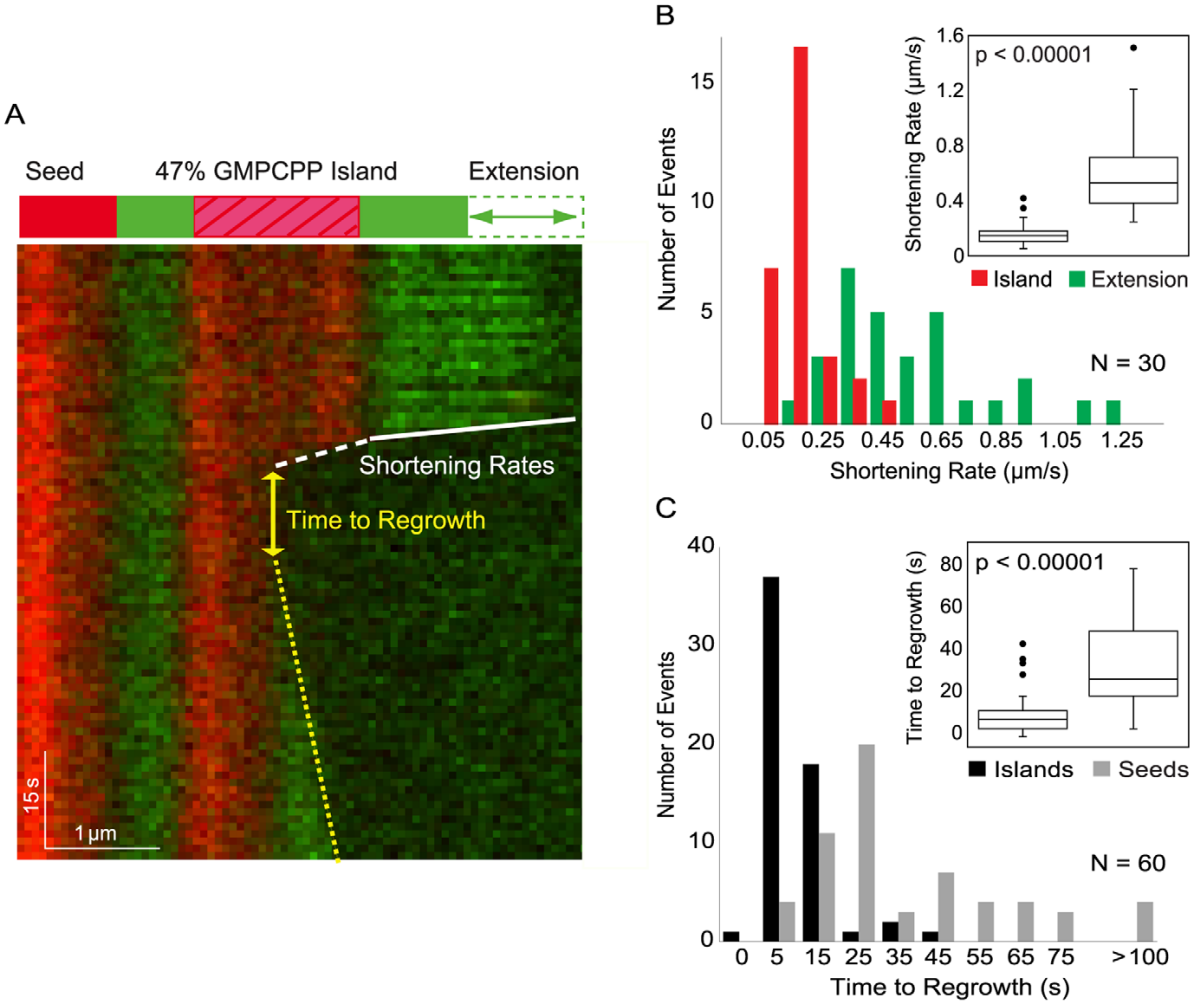
Island dynamics are distinct from GDP-tubulin extensions and GMPCPP seeds. (A) Shortening rates of GDP-tubulin extensions (white solid line) and GXP islands (white dashed line) and the time to microtubule regrowth (yellow double arrow measured from extrapolated yellow dotted line) were measured from kymographs of depolymerizing microtubules. (B) GXP islands shorten more slowly than GDP-tubulin extensions. Distribution of shortening rates measured for extensions and islands (*N* = 30). The inset shows a box-plot of the data, which has a median shortening rate of 0.49 µm/s for extensions and 0.16 µm/s for islands (*p*<0.00001, Wilcoxon rank-sum test). Only cases where extension and island shortening could both be measured in the same depolymerization event were analyzed. (C) Microtubules grow more readily from islands than from GMPCPP seeds. Time to regrowth was measured from kymographs of dynamic microtubules and was on average shorter from islands than from GMPCPP seeds. Median time to regrowth was 6 seconds for GXP islands (*N* = 60) and 27 seconds for GMPCPP seeds (*N* = 60) (inset, *p*<0.00001, Wilcoxon rank-sum test; outliers >100 are not shown).

The above results demonstrate that GXP islands promote microtubule rescue. However, we also wanted to investigate whether the GXP islands would give rise to microtubule shortening events distinct from standard tubulin catastrophe events. We used kymographs to measure the shortening rates of green GDP-tubulin extensions and their respective red GXP islands for 30 depolymerization events (Fig. 3A). For these events, the median shortening rate through extensions was 0.49 µm/s (*N* = 30), and the median shortening rate through islands was 0.16 µm/s (*N* = 30) (*p*<0.00001, Wilcoxon rank-sum test; Fig. 3B). Thus the GXP islands depolymerize at a significantly slower rate than the dynamic GDP-tubulin extensions, predicting that depolymerization may also slow down at natural sites of rescue.

We also compared the time to regrowth from GXP islands to the time to regrowth from GMPCPP seeds. As shown in Figure 3C, the median time to regrowth from the islands (6 s, *N* = 60) is considerably shorter than the time to regrowth from seeds (27 s, *N* = 60) (*p*<0.00001, Wilcoxon rank-sum test). For comparison, the corresponding rescue times for GTP tubulin have been reported to be ≤10 s [15]. Our findings therefore indicate that GXP islands may more closely correspond to GTP-tubulin rescue events than regrowth at GMPCPP seeds. For example, a new nucleation event may be necessary to allow for regrowth from a GMPCPP seed, while the structural configuration of a GXP island may be closer in structure and composition to a dynamic microtubule and can thus induce regrowth on shorter time scales.

In conclusion, our results show that rescue-promoting GXP islands can be reconstituted *in vitro*, providing a new tool for studying microtubule rescue. Interestingly, we observed that microtubule regrowth from these reconstituted islands occurred more readily than from GMPCPP seeds, suggesting that these islands may indeed behave more like naturally occurring GTP islands than GMPCPP seeds. Taken together, our findings support a model for rescue in which regions enriched in GTP-tubulin may be able to slow and stop microtubule depolymerization, akin to the “speed bumps” and “stop signs” previously put forth by Cassimeris [5].

## Materials and Methods

### Protein preparation

Tubulin was purified from porcine brains obtained from a local slaughterhouse (Gausepohl Schlachthof, Chemnitz) and labeled according to the standard protocols [16], [17]. GMPCPP microtubules were prepared as previously described [18]. The coding region of His-EB1 OmicsLink Expression Clone (GeneCopoeia) was cloned into a pETMM-11 vector. The recombinant fusion protein was expressed in *E. coli* (pRARE Cells) and purified using a Ni Sepharose column (HisTrap HP, GE Healthcare). The His-tag was removed by digestion with TEV protease. Protein concentration was determined using a Bradford assay and absorbance at λ = 280 nm.

### Assay Conditions

Silanization of cover glasses and preparation of flow-cells was previously described [19], [20]. The assay protocol for immobilization of microtubules in a flow-cell was previously described [21]. To create GXP islands, 15% rhodamine-labeled, 10% biotynylated GMPCPP seeds were immobilized on glass cover slips in flow chambers. The following was flowed into the chamber at ∼1–1.5 minute intervals:

- 7 µM 20% alexa488-labeled tubulin in Imaging Buffer supplemented with 1 mM GTP.
- 7 µM 10% rhodamine-labeled tubulin in Imaging Buffer supplemented with 100 µM GTP and 286 µM GMPCPP (for 74% GMPCPP) or 100 µM GTP and 100 µM GMPCPP (for 50% GMPCPP).
- 7 µM 20% Alexa488-labeled tubulin in Imaging Buffer supplemented with 1 mM GTP and 400 nM EB1.

The Imaging Buffer consisted of BRB80 supplemented with 50 mM KCl, 40 mM glucose, 40 mg/ml glucose-oxidase, 16 µg/ml catalase, 0.16 mg/ml casein, 1% DTT, and 0.01% Tween-20. For all the experiments an objective heater was used to warm the sample to 28°C.

### Imaging

The imaging setup utilizing TIRF was previously described [20]. Images were collected with Andor iXon3 and QuantEM: 512SC cameras on a Nikon Eclipse Ti microscope, using a Nikon Apo TIRF 100×/1.49NA objective and 1.5 or 2.5× optovar. Standard filter sets were used to visualize rhodamine and alexa-488 fluorescence. In measuring depolymerization speed, we only included traces in which the ends were clear enough to trace a straight line from which to calculate the slope (white lines shown in Fig. 3A). We estimate that the velocity-measurement uncertainties are smaller than the microtubule-to-microtubule variation shown in Figure 3B.

## Supporting Information

Information S1 **Estimate of the fraction of GMPCPP-tubulin in GXP islands based on a published experimental data.** We develop a model for incorporation of GXP-tubulin into microtubules, fit the model to previously published experimental data, and then use the parameters from the model to estimate the fraction of GMPCPP-tubulin in our GXP islands.
(DOCX)

Table S1 **The number of observed rescue events for two different GMPCPP/GTP-tubulin island compositions.**
(DOCX)

Movie S1 **Movie of microtubule growth and depolymerization (kymograph shown in** **Fig. 1B** **) showing rescue at the end of an island, rescue in the middle of the island, and depolymerization through the island (no rescue).** The seed length is 3 µm and the total movie time is 36 minutes.
(MP4)
