# Supplementary material for: Islands Containing Slowly Hydrolyzable GTP Analogs Promote Microtubule Rescues
Source: PLoS One. 2012 Jan 17;7(1):e30103. doi: 10.1371/journal.pone.0030103 (PMC3260198; doi:10.1371/journal.pone.0030103)
Supplement: Information S1 — Estimate of the fraction of GMPCPP-tubulin in GXP islands based on a published experimental data. We develop a model for incorporation of GXP-tubulin into microtubules, fit the model to previously published experimental data, and then use the parameters from the model to estimate the fraction of GMPCPP-tubulin in our GXP islands. (DOCX) [file pone.0030103.s001.docx]

## Supporting Information S1

Islands Containing Slowly Hydrolyzable GTP Analogs Promote Microtubule Rescues

Carolina Tropini, Elizabeth A. Roth, Marija Zanic, Melissa K. Gardner and Jonathon Howard

**Estimate of the Fraction of GMPCPP-tubulin in GXP Islands**

The fraction of GMPCPP-tubulin in the GXP islands can be estimated as follows.

The dissociation constants for binding of GMPCPP and GTP nucleotides to unpolymerized tubulin dimers in solution are given by:

$K_{C}=\frac{[Tb][C]}{[Tb\cdot C]}$, and $K_{T}=\frac{[Tb][T]}{[Tb\cdot T]}$,

respectively. Here, $[Tb]$ denotes the concentration of free tubulin dimers in solution; $[C]$ and $[T]$ the concentrations of unbound GMPCPP and GTP nucleotides; and $[Tb\cdot C]$ and $[Tb\cdot T]$ the concentrations of tubulin dimers in solution which are bound to GMPCPP and GTP nucleotides, respectively.

The ratio of tubulin dimers in solution bound to GMPCPP vs. GTP nucleotide is given by:

$r\equiv\frac{[Tb\cdot C]}{[Tb\cdot T]}=\frac{K_{T}}{K_{C}}\frac{[C]}{[T]}\approx\frac{K_{T}}{K_{C}}\frac{[C^{tot}]}{[T^{tot}]}$,

where we assumed that the concentration of bound nucleotides is well below the total concentration of nucleotides in solution, which is valid in our experimental conditions. (Note: the concentration of tubulin used was 7 µM, whereas the nucleotide concentrations were in the 100 – 300 µM range.)

Similarly, the ratio of GMPCPP-bound tubulin dimers incorporating into the microtubule lattice to that of GTP-bound tubulin dimers is given by:

$r^{*}\equiv\frac{[Tb^{*}\cdot C]}{[Tb^{*}\cdot T]}\approx\frac{k_{C}}{k_{T}}\frac{[Tb\cdot C]}{[Tb\cdot T]}$,

where * denotes polymerized tubulin; $k_{T}$ and $k_{C}$are association rate constants for incorporation of GTP-bound and GMPCPP-bound tubulin dimers into the microtubule lattice; and we make use of the fact that only a very small fraction of nucleotide-bound tubulin is in the polymer form. (Note: the typical amount of polymerized tubulin in our experimental conditions is in the nanomolar range.)

Therefore,

$r^{*}=\frac{k_{C}}{k_{T}}r=\frac{k_{C}}{k_{T}}\frac{K_{T}}{K_{C}}\frac{[C^{tot}]}{[T^{tot}]}\equiv\frac{K_{T}^{*}}{K_{C}^{*}}\frac{[C^{tot}]}{[T^{tot}]}$,

where $\frac{K_{T}^{*}}{K_{C}^{*}}$is the effective ratio of affinities of GMPCPP and GTP for the microtubule lattice.

The fraction of GMPCPP-tubulin in the GXP island lattice is now given by:

$$f=\frac{[Tb^{*}\cdot C]}{[Tb^{*}\cdot C]+[Tb^{*}\cdot T]}=\frac{r^{*}}{r^{*}+1}=\frac{\frac{K_{T}^{*}}{K_{C}^{*}}\frac{[C^{tot}]}{[T^{tot}]}}{\frac{K_{T}^{*}}{K_{C}^{*}}\frac{[C^{tot}]}{[T^{tot}]}+1}=\frac{\frac{[C^{tot}]}{[T^{tot}]}}{\frac{[C^{tot}]}{[T^{tot}]}+\frac{K_{C}^{*}}{K_{T}^{*}}}$$

Fitting the above relation to the experimental data from [10] (Fig. S1), we obtain the effective ratio of affinities:

$$\frac{K_{C}^{*}}{K_{T}^{*}}=3.87\pm0.25$$

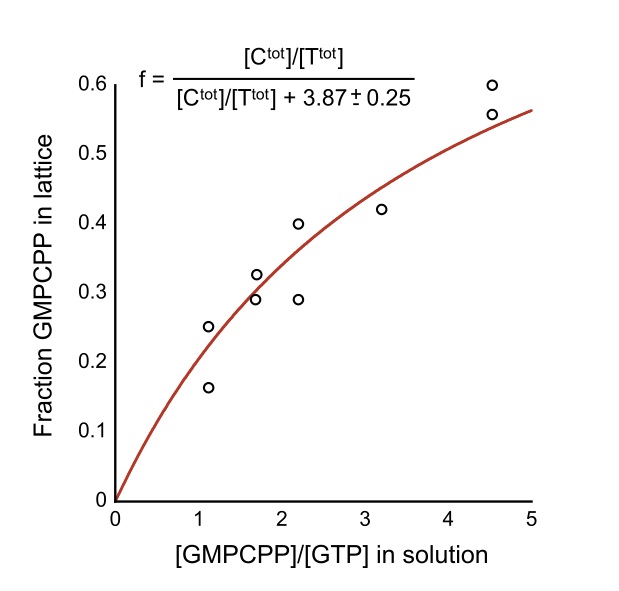


**Figure S1. Relative incorporation of GMPCPP-tubulin and GTP-tubulin into GXP islands** Experimental data from Caplow and Shanks, 1996, Figure 1 [10] was used to estimate the relative incorporation of GMPCPP- and GTP-tubulin into the lattices of growing microtubules as a function of the ratio of [GMPCPP] to [GTP] in solution. [GTP] = 100 μM. The data was fit to a Michaelis-Menten curve using the Levenberg-Marquardt algorithm in KaleidaGraph.

**
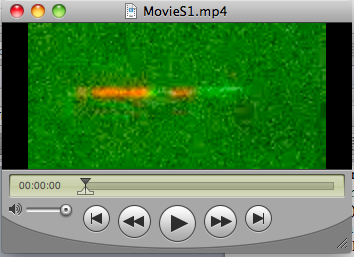
**

**Movie S1.** Movie of microtubule growth and depolymerization (kymograph shown in Fig. 1B) showing rescue at the end of an island, rescue in the middle of the island, and depolymerization through the island (no rescue). The seed length is 3 µm and the total movie time is 36 minutes.
