# Supplementary material for: Islands Containing Slowly Hydrolyzable GTP Analogs Promote Microtubule Rescues
Source: PLoS One. 2012 Jan 17;7(1):e30103. doi: 10.1371/journal.pone.0030103 (PMC3260198; doi:10.1371/journal.pone.0030103)
Supplement: Table S1 — The number of observed rescue events for two different GMPCPP/GTP-tubulin island compositions. (DOCX) [file pone.0030103.s002.docx]

## Supporting Table S1

Islands Containing Slowly Hydrolyzable GTP Analogs Promote Microtubule Rescues

Carolina Tropini, Elizabeth A. Roth, Marija Zanic, Melissa K. Gardner and Jonathon Howard

**Table S1. Probability of Rescue Events**

| 74% GMPCPP | | | | | |
| --- | --- | --- | --- | --- | --- |
| Island size bin mean (µm) | Number of rescue events | Number of no rescue events | Total number of events | Probability of rescue | 95% Confidence interval |
| 0.25 | 8 | 3 | 11 | 0.727 | 0.429-0.908 |
| 0.75 | 23 | 7 | 30 | 0.767 | 0.588-0.885 |
| 1.25 | 24 | 4 | 28 | 0.857 | 0.679-0.949 |
| 1.75 | 12 | 1 | 13 | 0.923 | 0.646-1.000 |
| > 2 | 8 | 0 | 8 | 1.000 | 0.628-1.000 |
| OVERALL | 75 | 15 | 90 | 0.833 | 0.742-0.898 |
| 50% GMPCPP | | | | | |
| OVERALL | 13 | 6 | 19 | 0.684 | 0.458-0.848 |

Number of observed rescue events for two different GMPCPP/GTP-tubulin island compositions. Results from 74% GMPCPP experiments are further divided by island size. The probability of rescue increases with island length and is higher for higher GMPCPP content. 95% confidence intervals were obtained using the Wald method.
